# Spastic Paraplegia Type 7 Is Associated with Multiple Mitochondrial DNA Deletions

**DOI:** 10.1371/journal.pone.0086340

**Published:** 2014-01-22

**Authors:** Iselin Marie Wedding, Jeanette Koht, Gia Tuong Tran, Doriana Misceo, Kaja Kristine Selmer, Asbjørn Holmgren, Eirik Frengen, Laurence Bindoff, Chantal M. E. Tallaksen, Charalampos Tzoulis

**Affiliations:** 1 Department of Neurology, Oslo University Hospital, Ullevål, Oslo, Norway; 2 University of Oslo, Faculty of Medicine, Oslo, Norway; 3 Department of Medical Genetics, Oslo University Hospital, Ullevål, Oslo, Norway; 4 Department of Clinical Medicine, University of Bergen, Bergen, Norway; 5 Department of Neurology, Haukeland University Hospital, Bergen, Norway; 6 Department of Neurology, Drammen Hospital, Vestre Viken Health Trust, Norway; University Nijmegen Medical Centre, Netherlands

## Abstract

Spastic paraplegia 7 is an autosomal recessive disorder caused by mutations in the gene encoding paraplegin, a protein located at the inner mitochondrial membrane and involved in the processing of other mitochondrial proteins. The mechanism whereby paraplegin mutations cause disease is unknown. We studied two female and two male adult patients from two Norwegian families with a combination of progressive external ophthalmoplegia and spastic paraplegia. Sequencing of *SPG7* revealed a novel missense mutation, c.2102A>C, p.H 701P, which was homozygous in one family and compound heterozygous *in trans* with a known pathogenic mutation c.1454_1462del in the other. Muscle was examined from an additional, unrelated adult female patient with a similar phenotype caused by a homozygous c.1047insC mutation in *SPG7*. Immunohistochemical studies in skeletal muscle showed mosaic deficiency predominantly affecting respiratory complex I, but also complexes III and IV. Molecular studies in single, microdissected fibres showed multiple mitochondrial DNA deletions segregating at high levels (38–97%) in respiratory deficient fibres. Our findings demonstrate for the first time that paraplegin mutations cause accumulation of mitochondrial DNA damage and multiple respiratory chain deficiencies. While paraplegin is not known to be directly associated with the mitochondrial nucleoid, it is known to process other mitochondrial proteins and it is possible therefore that paraplegin mutations lead to mitochondrial DNA deletions by impairing proteins involved in the homeostasis of the mitochondrial genome. These studies increase our understanding of the molecular pathogenesis of *SPG7* mutations and suggest that *SPG7* testing should be included in the diagnostic workup of autosomal recessive, progressive external ophthalmoplegia, especially if spasticity is present.

## Introduction

The hereditary spastic paraplegias (HSP) are clinically and genetically heterogeneous disorders characterized by the triad of progressive spastic paraparesis, hyperactive bladder and mild sensory dysfunction in the lower limbs [1]–[4]. Additional features include cerebellar ataxia, peripheral neuropathy, amyotrophy, extrapyramidal symptoms, cognitive impairment, deafness and visual impairment [1]. To date, over 50 loci have been mapped [5]–[7] and at least 25 genes identified, encoding proteins with a variety of functions including intracellular trafficking, mitochondrial metabolism and myelinization [8].

Spastic paraplegia 7 (SPG7) is an autosomal recessive disease caused by mutations in *SPG7* encoding the protein paraplegin. Paraplegin is a member of the AAA family of ATPases and contains both metallopeptidase and ATPase domains. It is located at the inner mitochondrial membrane and involved in processing mitochondrial proteins [9] and the assembly of the mitochondrial ribosome [10].

Over 50 pathogenic *SPG7* mutations have been reported including point mutations and large deletions affecting most of its 17 exons: missense mutations are the most frequent subgroup [11]. The prevalence of paraplegin mutations in sporadic or autosomal recessive HSP index cases ranges from 1.5% [12] to around 12% [6], [13]–[15].

Phenotypically, SPG7 is often a pure HSP, but cerebellar dysfunction and cerebellar atrophy on MRI are common [12], [16], [17]. Additional findings include upper limb hyperreflexia, sphincter dysfunction, spastic dysarthria, optical neuropathy, nystagmus, strabismus, decreased hearing, scoliosis, pes cavus, motor and sensory neuropathy, amyotrophy, blepharoptosis and ophthalmoplegia (Table 1) [1], [4], [12], [14]–[16], [18]–[22]. Onset is usually in adulthood, but ranges between 10–72 years [13], [16], [18], [21].

**Table 1:** Summary of the clinical features of four SPG 7 patients.

	Earlier studies	Patient AIV-5	Patient AIV-2	Patient BIII-2	Patient BIII-5
Age at onset	10–72 years	7 years	8 years	15 years	27 years
Age at examination		67 years	63 years	69 years	67 years
Ptosis	+	+	+	+	+
Progressive external ophthalmoplegia	+	+	+	+	+
Dysarthria	Spastic/cerebellar	Spastic	Spastic	Cerebellar	Cerebellar
Retinopathy	+	Pigmentation and atrophy	NA	–	NA
Dystonia	+	–	–	–	–
Cerebellar atrophy	yes	yes	yes	yes	NA
Upper limb hyperreflexia	+	+	+	+	+
Upper limb spasticity	+	+	+	+	–
Upper limb weakness	+	MRC 4	MRC 4	MRC 5	MRC 5
Lower limb hyperreflexia	+	+	+	+	+
Lower limb spasticity	+	+	+	+	+
Lower limb weakness	+	MRC 1–2	MRC 1–2	MRC 3–4	MRC 3–4
Plantar response inversion	+	+	+	+	+
Limb ataxia	+	+	+	+	+
Truncal ataxia	+	+	+	+	+
Urge incontinence	+	+	+	+	+
Decreased vibratory sense	+	+	+	+	+
Nystagmus	+	–	–	+	+
Cognitive impairment	+	+	NA	+	+
Dysphagia	+	–	NA	–	–
Decreased hearing	+	–	–	+	–
Pes cavus	+	–	–	+	–
Amyotrophy	+	+	+	–	–

The molecular pathomechanism of *SPG7* mutations remains unknown. Studies in skeletal muscle have shown evidence of mitochondrial respiratory chain (MRC) dysfunction in the form of cytochrome oxidase (COX) negative muscle fibers [13], [21], [23], [24] in some cases while another found none [16]. Fibroblast studies have shown evidence of reduced complex-I activity in some, but not in all patients tested [24], [25].

We identified a novel *SPG7* mutation in two Norwegian families presenting with ptosis and progressive external ophthalmoplegia (PEO) in addition to spastic paraparesis. Studies of the muscle mitochondrial DNA (mtDNA) showed evidence of multiple deletions, thus providing an explanation for the respiratory chain dysfunction often identified in this tissue and linking paraplegin to mtDNA homeostasis.

## Materials and Methods

### Ethics

Our study was approved by the Regional Committee for Medical and Health Research Ethics, South-Eastern Norway (ethical agreement n°129/04011). Written informed consent was obtained from all study participants.

### Patients

We studied two families (A and B), each with two affected siblings, from south eastern Norway. Muscle biopsy was available from an additional patient (CII-5) whose clinical and genetic features have been previously described [20].

**Figure 1:**
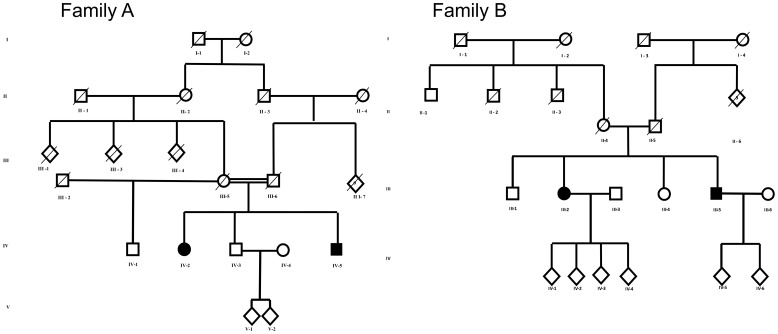
Pedigree of families A and B.

In Family A, the parents were first cousins, whereas in family B there was no known consanguinity (Figure 1). All patients underwent neurological examination according to a standard protocol that included Spastic Paraplegia Rating Scale (SPRS) and Scale for the Assessment and Rating of Ataxia (SARA) [26], [27]. Weakness was graded according to Medical Research Council (MRC) Scale for Muscle Strength. MRI, electrophysiological investigations and Intelligence Quotient (IQ) testing were performed in the index patients of the two families (Patients AIV-5 and BIII-2). IQ assessment was also done in patient BIII-5. Muscle biopsy was performed in patients AIV-5 and CII-5.

### Genetic Analyses

Genomic DNA was extracted from peripheral blood of patients and controls using standard techniques. Sequencing of *POLG1, POLG2* and *C10orf2* were performed in patient AIV-5. Sanger sequencing of the complete coding region of *SPG7* and MLPA (Multiplex Ligation-dependent Probe Amplification) analysis were performed in patient AIV-5 by Stichting Klinisch-Genetisch Centrum, Nijmegen, The Netherlands, using standard methods. Targeted mutation analysis was performed by Sanger sequencing in all patients and 192 controls (Table S1). Sequencing was done in an ABI 3730×l DNA analyzer (Life Technologies Corporation, Carlsbad, California) using ABI BigDye dye terminator cycle-sequencing kits (Life Technologies Corporation). Alignment of patients and controls sequences versus the February 2009 human reference sequence (GRCh37) was obtained using Mega software (http://www.megasoftware.net/mega4/mega.html) [28].

### Expression Studies

RNA was extracted from blood of patient AIV-5 and controls using a commercial kit (RNeasy Mini Kit, Qiagen) and converted to cDNA using High Capacity cDNA Reverse Transcription Kit (Life Technologies Corporation). In order to assess splice effects, cDNA from the patient and two controls was used to amplify exons flanking the mutation (Table S1). PCR products were subsequently run on an 1.5% agarose gel, Sanger sequenced and aligned using Mega software. SPG7 expression was assessed in blood leukocytes of patient AIV-5 and seven healthy controls by real time-PCR (RT-PCR) using SYBR Green JumpStart Taq ReadyMix chemistry (Sigma-Aldrich, Saint Louis, MO) and primers targeting SPG7 cDNA (Table S1). Reactions were run in triplicate on the ABI Real Time PCR 7900 HT Sequence Detector System (Life Technologies Corporation) and amplification levels were calculated as previously described [29]. Primers were designed using Primer 3 (http://frodo.wi.mit.edu/) [30].

### In silico Analyses

Conservation score for the mutated DNA base was evaluated using PhastCons and Genomic Evolutionary Rate Profiling (GERP) software available at the University of California Santa Cruz [31], [32]. Positive scores represent a substitution deficit and thus indicate that a site may be under evolutionary constraint. Negative scores indicate that a site is probably evolving neutrally.

Potential effects of the point mutation on protein structure and function was evaluated using the prediction softwares SIFT (Sorting Intolerant From Tolerant; http://sift.jcvi.org/www/SIFT_intersect_coding_submit.html) [33] and PolyPhen-2 (Polymorphism Phenotyping v2; (http://genetics.bwh.harvard.edu/pph2/) [34], [35].

### Muscle Biopsy

Open muscle biopsy was performed from the vastus lateralis muscle of patients AIV-5 at the age of 67 and CII-5 at the age of 43 years. The muscle sample was snap-frozen in isopentane cooled in liquid nitrogen and stored at −80^o^C. The muscle biopsies of a patient with a single mtDNA deletion (SD), a patient with multiple mtDNA deletions due to *POLG* mutations (MD) and 10 individuals with no mitochondrial or neuromuscular disease and a mean age of 37.1±16.5 years (range 18–62) at the time of the biopsy were used as controls.

### Histochemistry & Immunohistochemistry

Histological and histochemical studies were performed in the muscle of patient AIV-5, the two disease controls (MD, SD) and all healthy controls (n = 10). Serial 8 µm frozen sections were cut, air dried for 1 hour and stained with histochemical (HC) and immunohistochemical (IHC) methods. Sequential histochemical assay of cytochrome oxidase (COX) and succinate dehydrogenase (SDH) activity was performed as previously described [36]. For immunohistochemistry the sections were fixed for 15 min in cold acetone (4^o^C), rinsed in distilled water, washed in Tris-buffered saline (TBS) containing 0.1% Tween 20 (TBST) for 10 min, permeabilised in a graded methanol series containing hydrogen peroxide to inhibit endogenous peroxidase activity, washed in TBST for 5 min and incubated in optimal concentrations of primary antibodies for 1 hour at room temperature. Primary antibody binding was detected with a commercial kit (MACH 4 Universal HRP-Polymer Kit with DAB, Biocare Medical). Sections were washed in 2 changes of TBS for 5 min, incubated with a universal probe recognizing mouse and rabbit primary antibodies for 30 min, washed in 2 changes of TBS and incubated in a horseradish peroxidase (HRP) polymer for 30 min at room temperature. Sections were then washed in 3 changes of TBS for 5 min and visualized with 3,3-diaminobenzadine (DAB), counterstained in Mayer hematoxylin (Sigma Aldrich) for 15 sec, and the nuclei were blued in Scott tap water (Sigma Aldrich). Section analysis and cell counts were performed on a Leica light microscope (Leica Microsystems, GmbH Wetzlar, Germany) using Zeiss ZEN imaging software. Primary antibodies were used against respiratory complex-I 20 kDa subunit (NDUFB8) (diluted 1∶100), complex-II 70 kDa subunit (SDHA) (1∶1000), complex-III core-2 protein subunit (UQCRC2) (1∶500), complex-IV subunit CO1 (1∶400) and porin subunit VDAC1 (1∶2000). All antibodies were purchased from Abcam. The degree of complex deficiencies in skeletal muscle is expressed as a proportion of unstained (negative) fibres.

### MtDNA Studies

MtDNA studies were performed in muscle homogenate DNA of patients AIV-5 and CII-5, the two disease controls with single (SD) and multiple (MD) mtDNA deletions and the 10 healthy controls. Single fibre studies were performed in patient AIV-5, the two disease controls (MD, SD) and three healthy controls aged 26, 38 and 57 years at the time of the biopsy. Genomic DNA was extracted from muscle biopsy homogenate using a commercial kit (QIAamp DNA Mini Kit, Qiagen). Single muscle fibres were laser-microdissected from 20 µm frozen sections stained with COX/SDH histochemistry using a PALM microdissection microscope (Zeiss). Individual muscle fibres were lysed overnight in 15 µl of lysis buffer as previously described [37]. MtDNA deletions were detected in DNA from muscle homogenate using long-range PCR (LPCR) to amplify an 8.3 kb (8232–16496) fragment as previously described [38]. Quantification of total and deleted mtDNA was performed in muscle homogenate and single fibre lysates using real-time PCR to compare amplification within a rarely deleted area (*MT-ND1*) to that of a commonly deleted area (*MT-ND4*) as previously described [37], [39].

## Results

### Case Reports

The clinical features of the patients are summarized in Table 1.

**Family A.** *Subject AIV-5 (index)*. This man who is currently 67 years of age, developed progressive problems with coordination of upper and lower limbs and lower limb stiffness from the age of seven years. He walked with a stick from the age of 25 years and became wheelchair bound aged 39. Neurological examination at the age of two years, following a minor head injury, was unremarkable with the exception of a unilateral extensor plantar response. Examination at the age of 22 years after a traffic accident revealed failure of abduction of the left eye, hyperreflexia in all four extremities and Babinski sign. Follow-up at the age of 40 years revealed severe spastic paraparesis with bilateral Babinski sign, and in the upper limbs moderate weakness and hyperreflexia was present. There was bilateral ptosis and partial conjugate gaze palsy in all directions. He had symptoms of a neurogenic bladder, without incontinence. Electroencephalography (EEG), electromyography (EMG) and nerve conduction velocities (NCV) were normal.

Clinical examination by the authors at the age of 67 years showed a marked spastic paraparesis with severe muscle weakness. Moderate truncal ataxia was present with bilateral dysmetria and dysdiadochokinesia. It was not possible to assess cerebellar function in the legs due to weakness. There was a mild impairment of vibration sense at the ankles, otherwise proprioception, temperature and superficial sensation to touch and pin prick was normal. SARA-score was 29/40, and SPRS-score 43/52.

**Figure 2:**
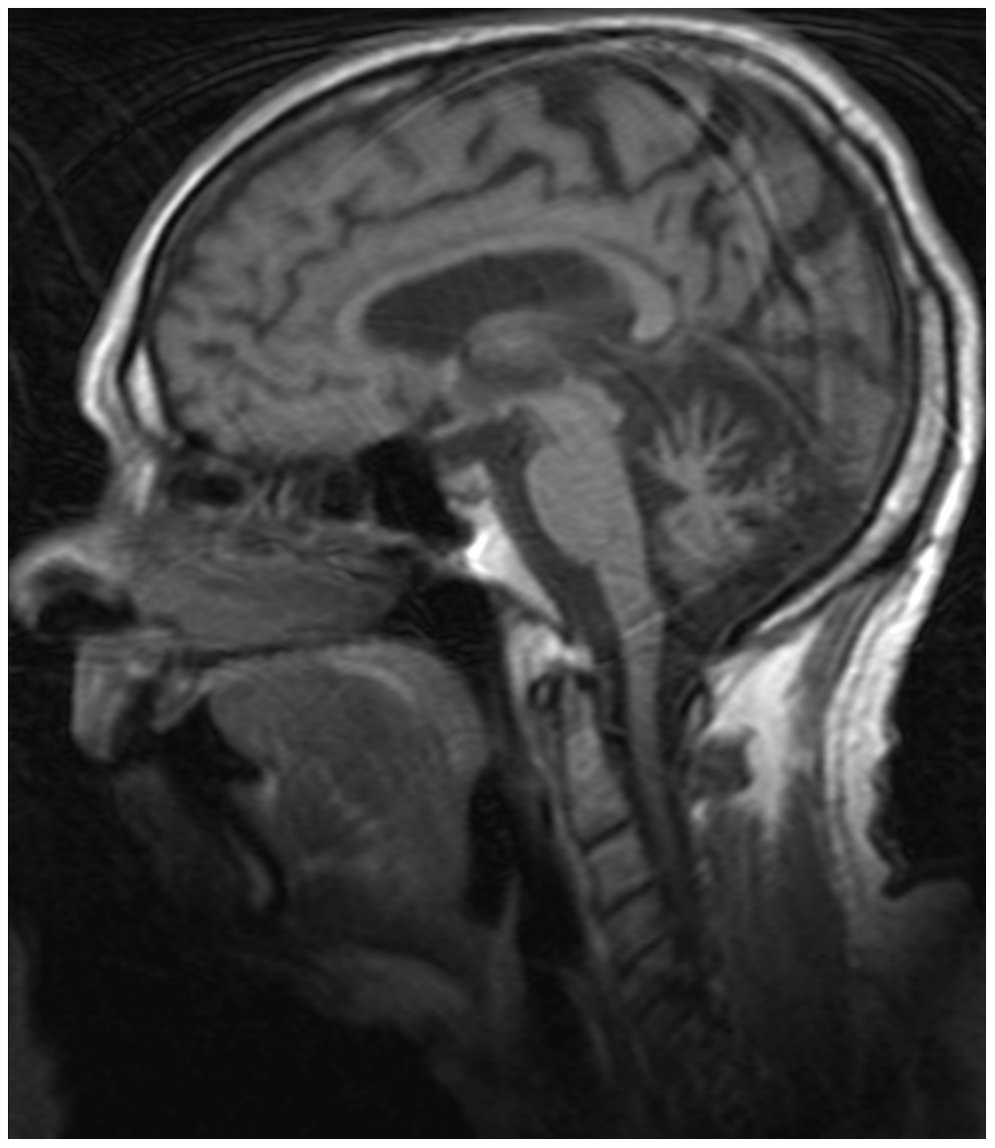
Brain MRI of patient AIV-5 showing cerebellar atrophy.

Ophthalmological examination showed a nearly complete external ophthalmoplegia with diminished oculocephalic reflex and severe ptosis with significant narrowing of the palpebral fissures (<4 mm) as well as continuous activation of the frontal muscle. There was increased retinal pigmentation of the right eye and paramacular atrophy in the left. Visual acuity was 0.4–0.6. Cognitive assessment with full scale 2-subtest Wechsler’s abbreviated scale of intelligence (WASI) [40] was suggestive of cognitive impairment with full IQ of 79, of which his verbal IQ was equivalent with the 32^nd^ percentile, and his executive IQ with the 41^st^ percentile. MRI of the brain revealed cerebellar atrophy and mild atrophy of the corpus callosum (Figure 2), whereas MRI of the spine and cord was normal. NCV showed a mixed, but primarily axonal sensorimotor peripheral neuropathy with low sensory amplitudes and normal conduction speed, and low motor amplitudes and delayed conduction speed. EMG showed a slight decrease in the recruitment pattern. Serum creatine kinase (CK) levels were normal.

*Subject AIV-2.* This 64-year old female developed progressive unsteadiness from the age of eight years. She experienced leg stiffness, but poor coordination and unsteadiness were her main complaints. Subsequently, she developed urge incontinence and mild dysarthria. She became wheelchair bound from the age of 43 years. Examination at the age of 63 years showed severe cerebellar ataxia in truncus and extremities. There was hyperreflexia in both upper and lower limbs and spastic paraplegia with a bilateral Babinski sign. She had external ophthalmoplegia with impaired vertical eye motility, slow horizontal saccades and impaired slow pursuit. In addition there was a moderate bilateral ptosis with the palpebral aperture measuring 5 mm [41]. Serum creatine kinase (CK) levels were normal.

*Subjects AIV-1* and *AIV-3* had no symptoms and normal neurological examination.

**Family B.** *Subject BIII-2 (index).* This 69 year old woman developed weakness in the left leg at the age of 15. From the age of 30 she developed progressive unsteadiness and lower limb stiffness followed by urinary urge incontinence. She underwent bilateral blepharoptosis surgery at the age of 61. Examination at the age of 62 showed spastic paraparesis, with retained ability to walk short distances with a walker. There were subtle cerebellar signs in the upper limbs and mild cerebellar dysarthria. Ophthalmological examination showed normal retinal and macular findings, but visual acuity was 0.3–0.5. MRI of the brain showed severe cerebellar atrophy, especially affecting the vermis. MRI of the spine and cord was normal.

Follow-up examination at the age of 69 revealed mild worsening of the spastic paraplegia.

SARA-score was 26/40, and SPRS-score 45/52. There was a moderate external ophthalmoplegia with reduced oculocephalic reflex and a severe ptosis with significant narrowing of the palpebral fissures (>4 mm). Cognitive assessment with full scale 2-subtest WASI was suggestive of cognitive impairment with full IQ of 78, of which her verbal IQ was equivalent with the 27^th^ percentile, and her executive IQ with the 44^th^ percentile.

*Subject BIII-5.* This man developed progressive gait unsteadiness from the age of 27 years. He lost unsupported gait in his mid-thirties, but is still able to walk with the aid of two sticks at the age of 67. Examination at the age of 67 revealed spastic paraparesis, dysarthria and mild upper limb ataxia. There was a mild impairment of vibration sense, but proprioception and superficial sensation were normal. He had bilateral ptosis with the palpebral aperture measuring 5 mm and external ophthalmoplegia which was complete for vertical gaze and moderate for horizontal movements. In addition, horizontal gaze showed slow saccades and saccadic smooth pursuit.

Cognitive assessment with 2-subtest WASI showed IQ 78, of which his verbal IQ was equivalent with the 30^th^ and his executive IQ with the 41^st^ percentile.

**Family C.** The clinical and genetic features of subject CII-5 have been previously described [20].

### Muscle Biopsy

**Figure 3:**
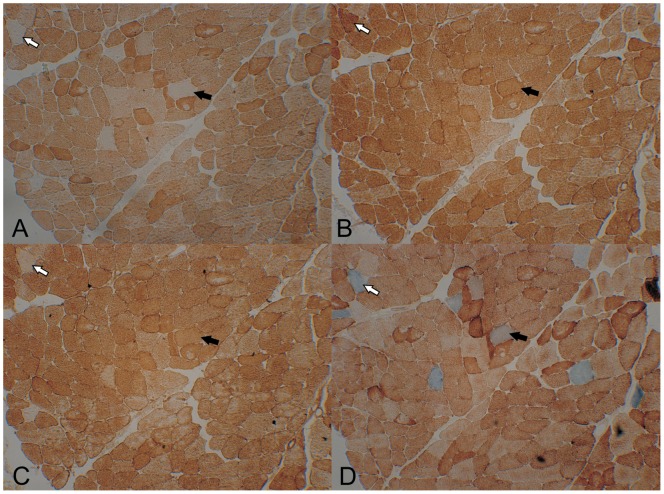
Immunohistochemistry in serial sections of the muscle of patient AIV-5. Immunohistochemistry for complex I (A), complex II (B), complex III (C) and COX/SDH histochemistry (D) in serial sections of the muscle of patient AIV-5. There are complex I, III and IV deficient fibres, but complex I deficiency is most pronounced. Arrows mark serial sections of the same muscle fibers stained for different complexes.

Routine muscle histology in the index patient of family A (Patient AIV-5) showed no ragged red fibres and was otherwise normal. IHC and HC showed deficiency of the respiratory complexes I (4.5%), III (3.5%) and IV (3.3%). Complex II and porin stained all fibres and no defects were seen in control muscle (Figure 3). Multiple complex deficiencies co-localized in the same fibres, but complex I loss was more widespread. Electron microscopy showed increased amount of fat vacuoles within the muscle fibres and was otherwise normal.

### Genetic Analyses

A novel homozygous point mutation c.2102A>C in *SPG7* (NM_003119.2) was found in the two affected siblings of family A and was heterozygous in unaffected subjects AIV-1 and AIV-3. The mutation segregated in the families and was absent in 384 chromosomes from 192 ethnic Norwegian controls. This is a missense mutation changing a highly conserved histidine in position 701 to a proline (p.H 701P) in the Pfam Peptidase domain (http://smart.embl-heidelberg.de/) of the protein (Figure S1). The mutation is located at the donor splicing site of exon 15, but amplification and sequencing of a cDNA segment spanning exons 13–17 showed no evidence of aberrant splicing (Figure S2). Real-time PCR analysis of cDNA from blood of patient AIV-5 did not detect significant differences in SPG7 expression in the patient compared to seven healthy controls (Figure S2).

*In silico* analysis of the mutation by PolyPhen-2 predicted no effects on protein stability with a score of 0.260 (sensitivity: 0.91; specificity: 0.88). SIFT prediction of the mutation also indicates the mutation to be tolerated with a SIFT-score of 0.21 and a median of 2.57.

In family B the same mutation was found compound heterozygous *in trans*, with the earlier described pathogenic in frame deletion c.1454_1462 (pArg485_Glu487del) [21].

Sequencing of *POLG1, POLG2* and *C10orf2* was normal in patient AIV-5.

### MtDNA Studies in Muscle

**Figure 4:**
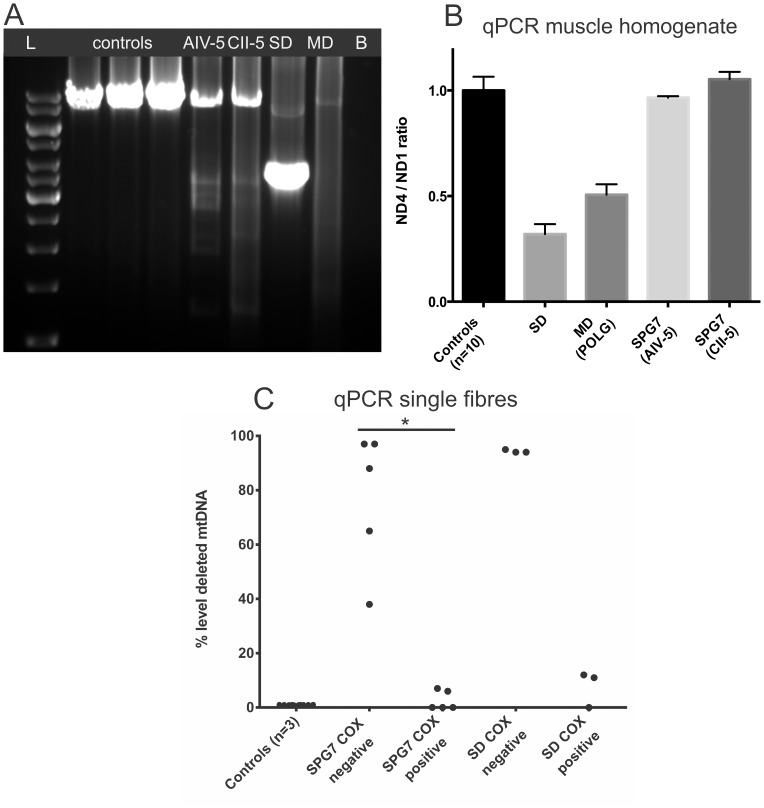
MtDNA studies in the muscle of two SPG7 patients and controls. MtDNA studies in the muscle of two SPG7 patients (AIV-5 and CII-2) and controls. Results from a patient with a single mtDNA deletion (SD) and a patient with multiple mtDNA deletions due to POLG mutations (MD) are also shown for comparison. A: LPCR of mtDNA shows multiple deletions in the two SPG7 patients. The ladder is 1 kb (GeneRuler). B: blank. B: qPCR of mtDNA in muscle homogenate shows no detectable deletions (ND4/ND1 ratio within the control range) in the SPG7 patients. Low ND4/ND1 ratios consistent with ∼60% and ∼50% deleted mtDNA are found in the patients with single and multiple mtDNA deletions respectively. Error bars mark standard deviations. C: Scatter plot showing the proportion of deleted mtDNA in microdissected COX-positive and COX-negative muscle fibres from an SPG7 patient (AIV-5) and a patient with single mtDNA deletion. Deletions reach significantly higher levels (38–97%) in the COX-negative, than in the COX-positive fibres (0–7%) of the SPG7 patient. No deletions are detected in single fibres from three healthy controls. Each dot represents data from a single fibre. COX: cytochrome-oxidase. *P = 0.008 (comparison by Mann-Whitney test).

Long-range polymerase chain reaction (LPCR) in muscle homogenate DNA showed a normal sized band at 8 kb together with several smaller bands consistent with the presence of multiple mtDNA deletions in both SPG7 patients examined (Figure 4A). No deletions were detected in muscle homogenate by real-time PCR which showed a normal ND4/ND1 ratio (Figure 4B). Single fibre analysis in patient AIV-5 showed that deleted mtDNA species segregated at significantly (*p* = 0.008) higher levels (38–97%) in COX-negative fibres than in in COX-positive fibres where they were absent or present at very low levels (0–7%). No deletions were detected in single fibres from three healthy controls (Figure 4C).

MtDNA quantification against the APP gene showed no significant changes in mtDNA content in the muscle of the patients compared to the mean of 10 healthy controls (data not shown).

## Discussion

Our findings elucidate novel aspects of the molecular pathogenesis associated with disease causing mutations in *SPG7*. We show that this disorder is associated with mtDNA damage in the form of multiple deletions that generate multiple respiratory complex deficiencies in skeletal muscle. This suggests that SPG7, like other complex diseases affecting the nervous system such as dominant optic atrophy 1 (DOA1), is linked to the maintenance of mitochondrial DNA. Mitochondrial DNA changes were found with two different *SPG7* mutations, the novel c.2102A>C and the c.1047insC reported previously [20]. MtDNA abnormalities have never been demonstrated in SPG7 and while respiratory chain dysfunction has been found [24], [25] the aetiology has been unclear.

Although multiple mtDNA deletions were clearly detected by LPCR in both patients, quantifying them by qPCR required analysis in single fibres. This is due to the fact that deleted mtDNA segregated in respiratory deficient cells of which there were fewer than 5% in the muscle biopsy. Uneven distribution of mtDNA defects in individual muscle fibres is a common phenomenon that has been reported with both single and multiple mtDNA deletions [39], [42].

The molecular mechanisms underlying the formation of mtDNA deletions in SPG7 remain unclear. Multiple mtDNA deletions commonly occur in disorders affecting either mtDNA homeostasis directly, such as mutations of *POLG* and *C10orf2* [38], or mitochondrial quality control including mutations of *OPA1* [43] and *MFN1* [44]. Although paraplegin is not known to be directly associated with the mitochondrial nucleoid, it is involved in the processing of other mitochondrial proteins. It is therefore possible that paraplegin mutations lead to mtDNA damage and respiratory deficiency by impairing the function of other mitochondrial proteins involved in either mtDNA replication itself or pathways of mitochondrial quality control.

The high levels of mtDNA deletion in COX-deficient fibres and the fact that respiratory deficiency in muscle selectively affected all three complexes with subunits encoded by mtDNA, but not complex II, suggest that this is indeed caused by mtDNA damage. Interestingly, complex I was more severely affected than III or IV. Selective complex I deficiency is seen with other disorders of mtDNA homeostasis including POLG and Twinkle associated disease [38], [45] and may have to do with the high number (seven) of complex I subunits encoded in mtDNA and/or with the fact that five of those subunits are encoded in the part of mtDNA commonly affected by deletions.

In spite of their overall low levels in the vastus muscle, it is possible that mtDNA deletions play an important role in the pathogenesis of SPG7. MtDNA defects of either primary or secondary etiology are known to segregate predominantly in clinically affected tissues. The extraocular muscles and nervous system, which are the primarily affected organs in this condition, may harbour significantly higher levels of mtDNA defects, but data from these tissues are currently unavailable. From studies performed in other disorders such as DOA1, it has been shown that mtDNA deletions can be found in tissue from unaffected organs, e.g. muscle, even if the major clinical symptoms and findings involve other organs, such as e.g. optic nerve [46], [47].

We also report a novel *SPG7* mutation. The c.2102A>C, p.H 701P segregated with the clinical phenotype and was absent in a large number of unrelated, healthy controls. This mutation changes a highly conserved amino acid situated within a catalytic metallopeptidase domain in paraplegin, where most of the known pathogenic mutations occur [9]. We believe, therefore, that the mutation is pathogenic. *In silico* analysis did not predict significant changes in protein stability, but as the mutation was homozygous (or compound heterozygous) in affected individuals, overall effects on protein function may have been underestimated.

Ptosis [20] and ophthalmoplegia [22] have been reported previously in SPG7. Our findings confirm that this is indeed not an uncommon presentation of this disorder and we suggest that *SPG7* gene analysis should be included in the diagnostic workup of autosomal recessive PEO, especially if spasticity is present. PEO is a common mitochondrial disease phenotype and associated either with primary mtDNA mutations or mutations of nuclear genes involved in mtDNA homeostasis, such as polymerase gamma (*POLG1* & *POLG2*) [48], [49], *C10orf2* [50], *ANT1* [51], *TK2* [52] and *RRM2B* [53] that cause secondary mtDNA changes including multiple deletions and/or depletion.

Our findings increase our understanding of the molecular pathogenesis of *SPG7* mutations and link paraplegin to mtDNA homeostasis.

## Supporting Information

Figure S1 **The conservation score for the mutated DNA base according to PhastCons and Genomic Evolutionary Rate Profiling (GERP) software available at the University of California Santa Cruz.** The mutated base and coded amino acid in different species are highlighted by a red rectangle. Histidine is highly conserved at the position affected by the mutation.
(DOCX)

Figure S2 **Functional studies of the novel c.2102A>C mutation in** ***SPG7.*** A: Gel electrophoresis of PCR products obtained from cDNA of the proband (P) and two controls (C1, C2) using primer pairs 1 (primers L2 and R1) and 2 (primers L2 and R2) as indicated. All PCR products are of expected size, and no additional fragments were detected. Primer information is given in Table 1, supplementary material. B: Expression levels of *SPG7* in leucocytes from the patient (dark bars) compared with the mean of seven controls (light bars). *SPG7* expression was assessed with three primer pairs (SPG7_1-3) and compared to three housekeeping genes (*HPRT, PPIB, HMBS*).
(DOCX)

Table S1 **A: Primers for PCR on genomic DNA and Sanger sequencing.** B: Primers for cDNA amplification to check the splicing. C: Primers for cDNA to check the expression level in peripheral blood.
(DOCX)
