# Supplementary material for: Spastic Paraplegia Type 7 Is Associated with Multiple Mitochondrial DNA Deletions
Source: PLoS One. 2014 Jan 22;9(1):e86340. doi: 10.1371/journal.pone.0086340 (PMC3899233; doi:10.1371/journal.pone.0086340)
Supplement: Figure S1 — The conservation score for the mutated DNA base according to PhastCons and Genomic Evolutionary Rate Profiling (GERP) software available at the University of California Santa Cruz. The mutated base and coded amino acid in different species are highlighted by a red rectangle. Histidine is highly conserved at the position affected by the mutation. (DOCX) [file pone.0086340.s001.docx]

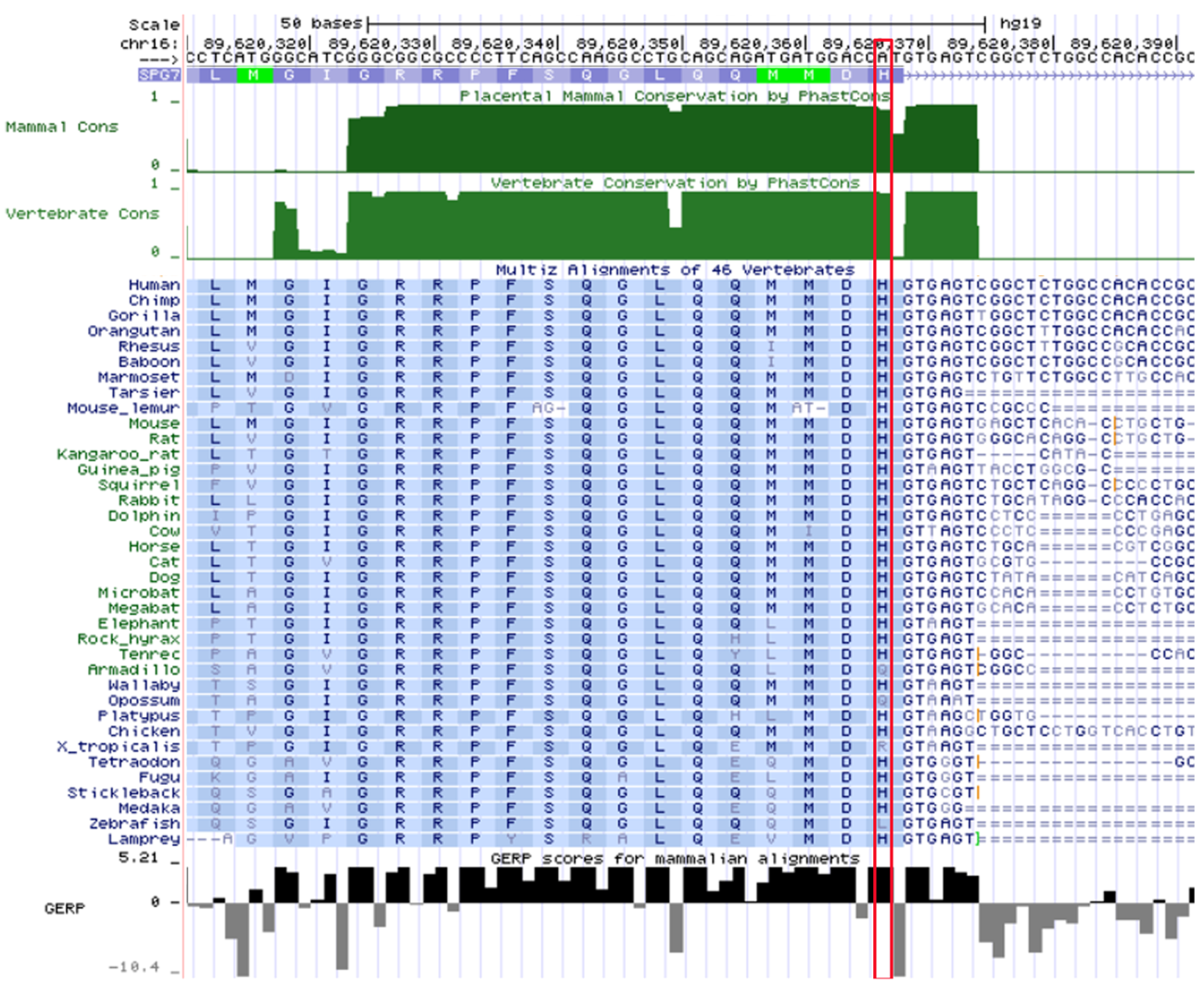


**Figure S1** The conservation score for the mutated DNA base according to PhastCons and Genomic Evolutionary Rate Profiling (GERP) software available at the University of California Santa Cruz. The mutated base and coded amino acid in different species are highlighted by a red rectangle. Histidine is highly conserved at the position affected by the mutation.
