# Supplementary material for: Spastic Paraplegia Type 7 Is Associated with Multiple Mitochondrial DNA Deletions
Source: PLoS One. 2014 Jan 22;9(1):e86340. doi: 10.1371/journal.pone.0086340 (PMC3899233; doi:10.1371/journal.pone.0086340)
Supplement: Figure S2 — Functional studies of the novel c.2102A>C mutation in SPG7. A: Gel electrophoresis of PCR products obtained from cDNA of the proband (P) and two controls (C1, C2) using primer pairs 1 (primers L2 and R1) and 2 (primers L2 and R2) as indicated. All PCR products are of expected size, and no additional fragments were detected. Primer information is given in Table 1, supplementary material. B: Expression levels of SPG7 in leucocytes from the patient (dark bars) compared with the mean of seven controls (light bars). SPG7 expression was assessed with three primer pairs (SPG7_1-3) and compared to three housekeeping genes (HPRT, PPIB, HMBS). (DOCX) [file pone.0086340.s002.docx]

**Figure S2** Functional studies of the novel c.2102A>C mutation in *SPG7*

**A**


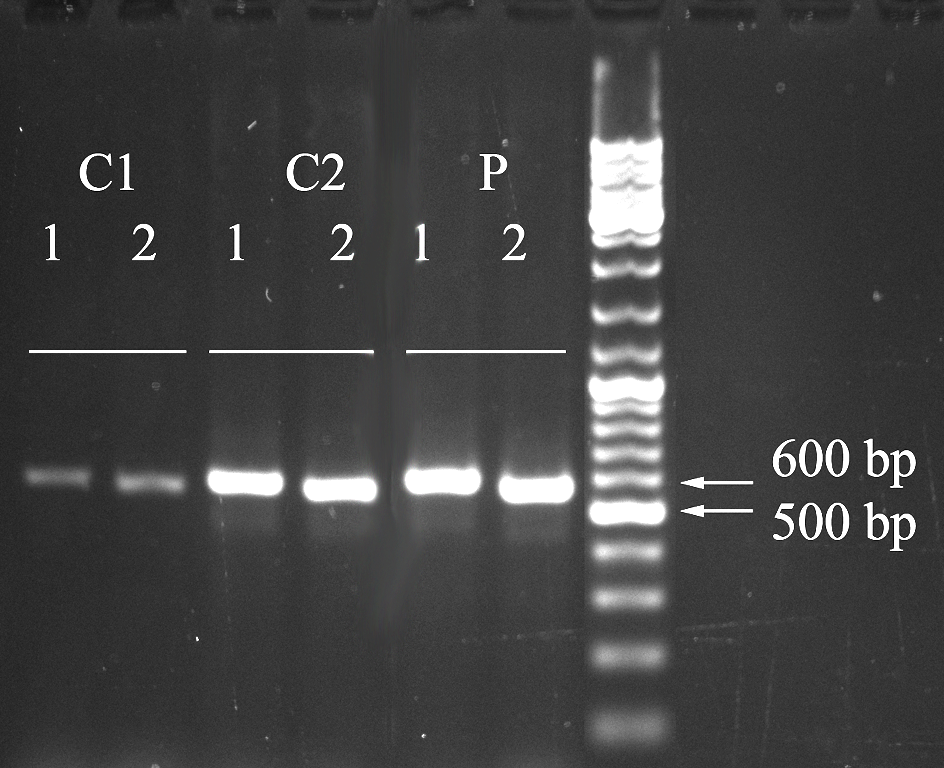


Gel electrophoresis of PCR products obtained from cDNA of the proband (P) and two controls (C1, C2) using primer pairs 1 (primers L2 and R1) and 2 (primers L2 and R2) as indicated. All PCR products are of expected size, and no additional fragments were detected. Primer information is given in Table 1, supplementary material.

B


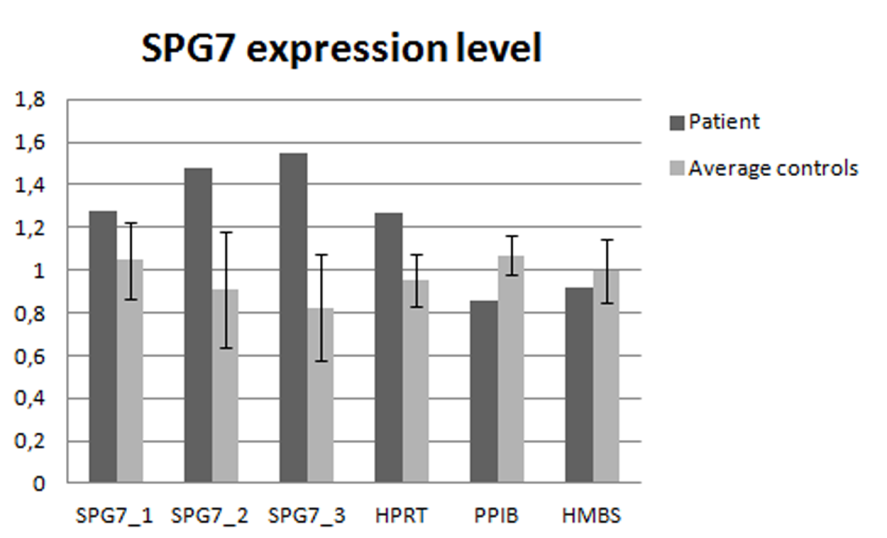


Expression levels of *SPG7* in leucocytes from the patient (dark bars) compared with the mean of seven controls (light bars). *SPG7* expression was assessed with three primer pairs (SPG7_1-3) and compared to three housekeeping genes (*HPRT, PPIB, HMBS*).
