# Supplementary material for: Spastic Paraplegia Type 7 Is Associated with Multiple Mitochondrial DNA Deletions
Source: PLoS One. 2014 Jan 22;9(1):e86340. doi: 10.1371/journal.pone.0086340 (PMC3899233; doi:10.1371/journal.pone.0086340)
Supplement: Table S1 — A: Primers for PCR on genomic DNA and Sanger sequencing. B: Primers for cDNA amplification to check the splicing. C: Primers for cDNA to check the expression level in peripheral blood. (DOCX) [file pone.0086340.s003.docx]

| **Table S1** |  |  |  |
| --- | --- | --- | --- |
| A |  |  |  |
| **Primers for PCR on genomic DNA and Sanger sequencing** | |  |  |
| Name | Sequence | Chr strand position in bp |  |
| SPG7 genomic L | AGGAAGGTCACCGGCATCGC | 16 + 89620219 89620238 |  |
| SPG7 genomic R | CTCACAGGCACACATCGGCCC | 16 - 89620659 89620679 |  |
|  |  |  |  |
| B |  |  |  |
| **Primers for cDNA amplification to check the splicing** |  |  |  |
| Name | Sequence | Chr strand position in bp/comment | Exon |
| Splicing SPG7_L1 | GCAGGGACTGCCAAAAAG | intron spanning | Exon 12-Exon 13 |
| Splicing SPG7_R1 | CTGGGTCTCTTCGGTCTCCT | 16 - 89623440 89623459 | Exon 17 |
| Splicing SPG7_L2 | AGGCCGTGATGAAGGTCTC | intron spanning | Exon 13-Exon 14 |
| Splicing SPG7_R2 | CTCGCCCAAGTCCTGTTTCT | 16 - 89623419 89623438 | Exon 17 |
|  |  |  |  |
| Primers combination | Expected PCR product (bp) | Exon |  |
| L2*+R1 | 581 | Exon 13-Exon 14 |  |
| L2*+R2 | 560 | Exon 17 |  |
|  |  |  |  |
| C |  |  |  |
| **Primers for cDNA to check the expression level in peripheral blood** | |  |  |
| RT-PCR SPG7_1f | CCGGCTTCTCCAACACGGA | 16 + 89598985 89599004 |  |
| RT-PCR SPG7_1r | GTCTTTCCTCTGAGCTTCCTCAT | 16 - 89603208 89603230 |  |
| RT-PCR SPG7_2f | CCATGTCCGGCTTCTCCAACA | 16 + 89598979 89599000 |  |
| RT-PCR SPG7_2r | CTCTGAGCTTCCTCATGGTCCC | 16 - 89603202 89603223 |  |
| RT-PCR SPG7_3f* | AGGCCGTGATGAAGGTCTC | intron spanning |  |
| RT-PCR SPG7_3r | CTTGGTGAAGAGGTGCTGGT | 16 - 89619445 89619467 |  |
|  |  |  |  |
| * same primer sequence |  |  |  |
